# Vitamin D status and its correlation to depression

**DOI:** 10.1186/s12991-022-00406-1

**Published:** 2022-08-18

**Authors:** Bashir khan, Huma Shafiq, Seyyedha Abbas, Summeira Jabeen, Sikandar Ali Khan, Tayyaba Afsar, Ali Almajwal, Nawaf W. Alruwaili, Dara al-disi, Sultan Alenezi, Zahida Parveen, Suhail Razak

**Affiliations:** 1 Institute of Basic Medical Sciences, Khyber Medical University Peshawar, Peshawar, Pakistan; 2 Institute of Cellular Medicine, Newcastle University Medical School, Newcastle University United Kingdom, Newcastle, England; 3 Department of Community Health Sciences, College of Applied Medical Sciences, King Saud University, Riyadh, Saudi Arabia; 4 University of Iceland, 101, Reykjavik, Iceland

**Keywords:** Vitamin D, Beck depression inventory, Tyrosine hydroxylase and depression

## Abstract

**Background:**

Vitamin D can influence more than 200 genes in various tissues showing its credibility among the fat-soluble vitamins. Vitamin D deficiency is directly proportional to major clinical conditions such as cardiovascular diseases, diabetes, malignancy, and multiple sclerosis. This study was conducted to determine the vitamin D level of individuals and its association with depression.

**Methods:**

Vitamin D levels of 100 healthy and 100 depressed subjects were determined. The isolated subjects were screened on the Beck Depression Inventory (BDI) scale and divided into three groups according to their age. Group-I comprised subjects of age 20 years and below, Group-II included subjects of age 21 to 60, and Group-III comprised subjects of ≥ 61 years of age. A sufficient level of vitamin D in normal subjects was noted, while mild deficiency of vitamin D status was observed in depressed subjects.

**Results:**

Our study has reported a higher percentage of vitamin D deficiency in the Peshawar region. The results of our study indicated that depression was common in individuals having vitamin D deficiency.

**Conclusions:**

The study showed a very high frequency of vitamin D deficiency in subjects with depression in Peshawar, Pakistan. The deficiency of vitamin D was observed more in females as compared to males. Further studies should explicate whether the highly widespread vitamin D deficiency could be cost-effectively treated as part of preventive or treatment interventions for depression.

**Supplementary Information:**

The online version contains supplementary material available at 10.1186/s12991-022-00406-1.

## Background

Vitamin D is also known as a secosteroid hormone known for its vital role in maintaining the normal function of bones. Research regarding the specific role of vitamin D in the immune system has been discovered. Vitamin D can influence more than 200 genes in various tissues, showing its credibility among the fat-soluble vitamins. Vitamin D deficiency is directly proportional to primary clinical conditions such as cardiovascular diseases, diabetes, malignancy and multiple types of scleroses [25]. Therefore, clinicians recommend large intake of vitamin D in the Diet so as to prevent these significant clinical conditions [22, 31]. Vitamin D deficiency exists throughout the world in various populations, including children, adults, both male and female (pregnant and lactating), and those who often avoid sunlight exposure. It is worth mentioning that individuals who have darkly pigmented skin are more prone to vitamin D deficiency [10].

Food is a limited source of vitamin D. Hence, overcoming the deficiency of vitamin D through food will not be sufficient. However, vitamin D supplements could be used to control its deficiency, but their efficacy is inconsistent and variable [10]. It has been noticed that vitamin D level is low in those individuals who have a mood disorder, and its mechanism of action has been noticed in causing depression [44, 52]. Vitamin D synthesis in the fair skin is enormously fast and significant even after a few minutes of exposure to sunlight [24]. Incidental sun exposure is the major and prominent source of circulating vitamin D [23, 43]. Upon exposure to sunlight in the summer season, Fair Skin can produce about 20,000 IU of vitamin D in less than 30 min [26]. A study conducted in the US in 89 different geographical locations on a large population suggested that the incidence of depression is greater in those people who have deficient vitamin D levels instead in those who have normal vitamin D levels [20].

Vitamin D has a key role in preventing rickets in children and reduces the risks of cancer, multiple sclerosis, and bacterial infections [21, 45]. Vitamin D deficiency leads to diabetes mellitus; its deficiency causes a decrease in microglial inflammatory function leading to increased brain infections [3, 29, 41. Zittermann et al. in 2003 and McCann and his colleagues in 2008 have revealed a significant correlation between vitamin D deficiency and brain dysfunctions [35, 54]. Vitamin D has a crucial role in developing a normal brain, while its deficiency is associated with morphological changes, such as enlarged ventricles and decreased cortical thickness [2, 18]. For about 30 years, a complicated interaction has been described between neuroinflammation, immune activation and modifications in brain circuits associated to depression and anxiety [34]. Therefore, vitamin D is known for regulation of innate immunity, both as a transcription and growth factor by interrelating with surface receptors in diverse immune cells [5]. Vitamin D is, therefore, associated with its ability to regulate both immune responses of peripheral and central nervous systems [46].

The antimicrobial properties of vitamin D has been described as its first immune-related properties, but it is also involved in the modulation of both innate and adaptive immune reactions [5]. In this perspective, depression and anxiety are often related with a low-grade inflammatory significance and peripheral increase in acute-phase proteins and inflammatory cytokines [17].

It is observed that vitamin D regulates the gene expression for one of the essential enzymes Tyrosine Hydroxylase, which is involved in synthesizing dopamine and norepinephrine. These neurotransmitters are famous for their role in depression and mood disorders [40]. Vitamin D maintains physiological functions, such as calcium homeostasis, membrane permeability and axonal conduction, and neurotransmission [11]. Vitamin D stimulates the receptors in those regions of the brain concerned with the regulation of emotion and behavior, such as the limbic system, cortex, and cerebellum. It also stimulates the release of neurotrophin, which has an important role in the regulation of neuronal development [39].

The ideal level of serum 25(OH) D levels lies between 100 and 150 nmol/L. Below 50 nmol/L are related to vitamin D deficiency; between 50 and 75 nmol/L levels are associated with moderate vitamin D deficiency. The criteria for interpretation of vitamin D values are divided into four main categories: vitamin D deficiency (less than 20 ng/mL), vitamin D insufficiency (21–29 ng/mL), vitamin D sufficiency (equal to or more than 30 ng/mL), vitamin D intoxication (more than 150 ng/mL) [12].

Emerging data from mouse models and human findings proposed that vitamin D showed potent immunosuppressant activity and might stimulate proinflammatory cytokines, for example IL-6 in the brain [49]. Vitamin D has numerous effects on the CNS to act as mood modulator, apart from the wide distribution of vitamin D receptors in brain regions closely implicated in depression and anxiety disorders pathophysiology. Though, further studies are required to clarify the mechanisms associated with mood improvement and to find out the groups of patients who might be benefited from vitamin D supplementation [5].

Mukesh and his colleagues determine the vitamin D level in the people of Pakistan [8]. This study was conducted to determine the vitamin D level of individuals with depression and the relationship of vitamin D with depression. Menon et al. showed low vitamin D levels in the population with depression [36]. Unfortunately, the issue of vitamin D level deficiency has been ignored in Pakistan. Hence, we identified the conclusion with the objective to find the significance of vitamin D level with depression.

## Methods

### Participants

A total of 200 subjects, including 100 healthy individuals and 100 depressed individuals (outpatients), were selected from various areas of Peshawar, Pakistan, at Cantonment Board Hospital Peshawar, Khyber Teaching Hospital Peshawar, and Lady Reading Hospital Peshawar. The selected individuals between 20 and below to 60 and above 60 years, were properly screened on BDI scale. Informed consent was taken from all participants included in the study. The study protocol was approved by the ethics committee in the Shaheed Benazir Bhutto Women University, Peshawar, Khyber Pakhtunkhwa, Pakistan. Studies reported in the manuscript fully meet the criteria for animal studies specified in the ACS ethical Guidelines.

### Study protocol

After taking their consent, all the participants were screened for inclusion in the study. Their blood samples were collected properly to determine their vitamin D level.

### Study design (descriptive)

All the necessary relevant information to the study participants was collected with informed written consent from the institutional review board and approved criteria for survey. Information about their vitamin D level and depression were compared to establish a relation between the two.

### Inclusion criteria

Participants of varied age with vitamin D deficiency and mild to severe depression were included in the study.

### Exclusion criteria

Study participants with chronic diseases such as liver and renal diseases, malabsorption syndrome, and other diseases that can affect the level of vitamin D were excluded from the study.

### Sampling technique

#### Collection of blood samples

Blood samples (3 mL) were collected from subjects, labelled, and stored in vials at 4°C. The tubes were centrifuged (Megafuge 1.0, Heraeus Sepatech) at 5000 rpm for 10 min. The blood serum was analysed for vitamin D level.

#### Determination of vitamin D level

For vitamin D determination, serum was separated. The serum level of vitamin D is monitored in Architect Plus, Abbott. The recorded data was noted automatically and further processed on the instrument CI 4100, where vitamin D level was determined and reported in ng/mL. The reference ranges are given in Additional file 1: Table S1 [19].

#### Identification of depressed and normal subjects

Depressed and normal subjects were initially identified by examining the patients for their mood and history. After preliminary identification of depressed subjects, their level of depression was further measured.

#### Estimation of level of depression

The level of depression in all the 100 depressed patients identified was determined using BDI scale described by Beck et al. [7]. BDI questionnaire contains 21 groups of statements necessary for estimating the depression level of subjects. Thus, in a typical experimental procedure, the most appropriate statement with all three groups were encircled by getting the history of the depressed subjects. The filled questionnaire was scored, and the level of depression was determined from the score as shown in Additional file 1: Table S2 [1].

## Results

To find the relation between vitamin D level and depression, the vitamin D levels of 100 healthy subjects and 100 depressed subjects were determined, as shown in Table 1. They were divided into three groups according to their age, i.e., Group-I comprised subjects below 20 years and below 20 years, Group-II included subjects between 21 and 60 years, and Group-III comprised of subjects above 61 years and above 61 years, and observation has been recorded, respectively.

**Table 1:** Comparative vitamin D level of total normal (100) and total depressed (100) subjects determined in nanogram per milliliter (ng/mL) given as mean ± SEM

S. No	Parameters	Subjects		*p* value
		Normal (ng/mL)	Depressed (ng/mL)	
1	Mean	46.34	23.60	< 0.05
2	Standard deviation	39.1	11.9	
3	Standard error of mean	3.91	1.2	
4	Coefficient of variance (%)	84.30	50.42	

*P* ≤ 0.05 = significant; *P* > 0.05 = non-significant

### Vitamin D profile of 100 normal subjects (males and females)

Table 2 shows the age and vitamin D data obtained for 100 normal male and female subjects. The mean ± SEM of the age of the normal male of 59 subjects was 39.28 ± 2.27 and the coefficient of variation (CV) of age was found as 44.42%. While the mean ± SEM of vitamin D level was found as 34.65 ± 3.84 and 84.84% CV.

**Table 2:** Age vitamin D level of normal male (59) and female (41) subjects determined in nanogram per milliliter (ng/mL) given as mean ± SEM

S. No	Parameters	Males (59)		Females (41)		*p* value	*P* value Vitamin D
		Age (years)	Vitamin D ng/mL	Age (years)	Vitamin D ng/mL	Age	
1	Mean	39.28	34.65	45.82	61.45	< 0.05	< 0.05
2	Standard deviation	17.45	29.4	14.56	45.86		
3	Standard Error of mean	2.27	3.82	2.3	7.16		
4	Coefficient of variance (%)	44.42	84.84	31.79	74.62		

*P* ≤ 0.05 =  significant; *P* > 0.05 = non-significant

Similarly, the age and vitamin D data of all normal female 41 subjects revealed that the mean ± SEM of age is 45.82 ± 2.3 with 31.79% CV, while 61.45 ± 7.16 was the mean ± SEM of vitamin D level and CV was observed 74.62%. The *p* value of age (*p* < 0.05) and *p* value of vitamin D (*p* < 0.05) are given.

### Age, vitamin D and depression profile of 100 subjects with depression (male and female)

Table 3 shows the age, vitamin D, and depression profile of 41 male subjects with depression. The data given in the table indicated the mean ± SEM of age is 38.87 ± 2.089, and 34.41% is the CV. The mean ± SEM of vitamin D level given is 24.17 ± 1.32 with a 34.96% CV. The mean ± SEM of depression level on the BDI scale given in the table is 25.8 ± 1.49 with a 37.17% CV.

**Table 3:** Age vitamin D level and BDI score of the male (41) and female (59) patients determined in nanogram per milliliter (ng/mL) given as the mean ± SEM

S. No	Parameters	Males			Females			*p* value	
		Age (years)	Vitamin D, ng/mL	BDI score	Age (years)	Vitamin D, ng/mL	BDI score		
1	Mean	38.87	24.17	25.8	45.5	23.36	28.06	< 0.05	> 0.05
2	Standard deviation	13.37	8.45	9.59	14.36	13.95	7.26		
3	Standard error of mean	2.09	1.32	1.49	1.87	1.81	0.95		
4	Coefficient of variance (%)	34.41	34.96	37.17	31.56	59.76	25.86		

*P* ≤ 0.05 = significant *P* > 0.05 = non-significant

Table 3 presents the age, vitamin D, and depression profile of all 59 female subjects with depression. The data obtained demonstrated the mean ± SEM of age as 45.5 ± 1.87 and 31.56% CV. The mean ± SEM of vitamin D level given is 23.36 ± 1.81 and 59.76% CV. The mean ± SEM of depression given is 28.06 ± 0.95, and 25.86% is the CV. The *p* value of age (*p* < 0.05) and *p* value of vitamin D was (*p* > 0.05) are given. The *p* value of age is significant, while that vitamin D is non-significant.

### Depression and vitamin D profile of subjects of 20 years and below (Group-I)

In Group-I (Table 4), subjects of 20 years and below comprising of 10 normal and 4 subjects with depression were evaluated for their vitamin D and depression level. For the 10 normal subjects, the mean ± SEM of age found was 15.9 ± 0.6 with a 12.01% CV. For vitamin D level, the mean ± SEM was calculated as 17.67 ± 0.56, and the CV was found as 9.91%.

**Table 4:** Age vitamin D level of normal and depressed along with BDI score of subjects 20 years of age and below of (Group I) given as the mean ± SEM

S. No	Parameters	Normal subjects		Depressed subjects			*P* value	*p* value
		Age	Vitamin D (ng/mL)	Age	Vitamin D (ng/mL)	BDI score	Age	vitamin D
1	Mean	15.9	17.67	17.75	23.2	19.0	> 0.05	> 0.05
2	Standard deviation	1.91	1.76	2.5	2.12	6.21		
3	Standard error of mean	0.6	0.56	1.23	1.06	3.1		
4	Coefficient of variance (%)	12.01	9.91	41.08	9.14	32.7		

*P* ≤ 0.05 = significant; *P* > 0.05 = non-significant

The depression and vitamin D profile of the 4 depressed subjects (Table 4) of this group showed mean ± SEM of vitamin D level as 23.2 ± 1.06, and 9.14% was recorded as the CV. The mean ± SEM of BDI score was calculated as 19 ± 3.1, and of age was calculated as17.75 ± 1.43. The *p* value of age (*p* > 0.05) and *p* value of vitamin D (*p* > 0.05) are non-significant.

### Depression and vitamin D profile of subjects between 21 and 60 years (Group-II)

Table 5 discusses the statistics of all the 70 normal subjects. The mean ± SEM of the age of 40 normal subjects determined was 39.18 ± 5.83 with a 14.9% CV. The mean ± SEM of vitamin D level of the 40 normal subjects was calculated as 44.51 ± 4.21 ng/mL, and the CV was 79.16%.

**Table 5:** Age vitamin D level of normal and depressed subjects of age 21 to 60 years of both sexes belonging to (Group II) given as the mean ± SEM

S. No	Parameters	Normal subjects		Depressed subjects			*p* value	*p* value
		Age	Vitamin D (ng/mL)	Age	Vitamin D (ng/mL)	BDI score	Age	Vitamin D
1	Mean	39.18	44.51	39.35	24.1	26.78	> 0.05	< 0.05
2	Standard deviation	48.79	35.24	9.78	12.89	8.6		
3	Standard error of mean	5.83	4.21	1.09	1.44	0.96		
4	Coefficient of variance (%)	14.87	79.16	24.85	53.48	32.11		

*P* ≤ 0.05 = significant; *P* > 0.05 = non-significant

For the group of age 21–60 years, the mean ± SEM of ages of all 80 subjects with depression were statistically determined as 39.35 ± 1.09, and the CV was calculated as 24.85%. The mean ± SEM of vitamin D level of all subjects was statistically calculated as 24.1 ± 1.44 ng/mL. The CV was determined as 32.1%, respectively, while 26.78 ± 0.96 as the mean ± SEM of depression (Table 5). The *p* value of age (*p* > 0.05) is non-significant and *p* value of vitamin D (*p* < 0.05) is significant.

Among all the depressed subjects, the vitamin D levels of 16 subjects (20%) were found normal, while vitamin D of 40 subjects (50%) was mildly deficient, and 24 subjects (30%) with depression were severely deficient in vitamin D level.

The relations between vitamin D and depression level in subjects with depression of age group 21 to 60 years of age have been summarized in Table 5. A total of 80 subjects were selected which were declared as depressed after examining them on the BDI scale.

All subjects with depression were scrutinized thoroughly on BDI scales to find their level of depression. It was found that 23 (28.7%) of them were mildly depressed, 19 subjects (23.7%) with moderate depressed and 38 subjects (47.5%) were found severely depressed. The moderate depressed 19 subjects (23.7%) were also examined for their vitamin D level, and significant results were found, 6 subjects (31.57%) had a severe deficiency of vitamin D, 10 subjects (52.6%) were found with mild deficiency, and only 3 subjects (15.7%) were having normal levels of vitamin D.

The other 38 subjects (47.5%) who were found as severely depressed on the BDI scale were also examined for their vitamin D level showed that 15 subjects (39.4%) had a severe deficiency of vitamin D, 16 subjects (42.1%) were found with moderate deficiency, and only 7 subjects (18.4%) had normal levels of vitamin D. In this age group an interesting relation between vitamin D and depression was noticed in the subjects. The results indicated the decrease in vitamin D levels was observed with increasing severity of depression calculated by the BDI scale.

The cumulative correlation between vitamin D and depression levels of all the mild, moderate, and severely depressed subjects, vitamin D levels of 20 subjects (25%) were found normal. A total of 28 subjects (35%) among the patients with depression showed a mild vitamin D deficiency, and 32 subjects (40%) with depression showed severe deficiency in their vitamin D levels. Moreover, the mean vitamin D of all the subjects with depression was found to be 24.1 ± 1.44 ng/mL, which is at the lower limit of mild deficiency and close to the upper margin of severe deficiency of vitamin D level with 53.48% CV.

Statistical analysis of BDI score of depression showed mean ± SEM, BDI score was 26.78 ± 0.96, which is the upper limit of moderate depression and approaching the least limits of severe depression. A 30.02% variation was recorded from the upper limit of the moderate depression showing 70% depression in the depressed subjects as at the upper level of moderate depression (Table 5).

The work was further continued to determine the relationship between the vitamin D level of normal and subjects with depression of age 21–60 years (Table 5). In the normal subjects, the mean ± SEM of vitamin D level was found to be 44.51 ± 4.21 ng/mL, and CV calculated was, respectively. While the mean of vitamin D level of subjects with depression was 23.15 ng/mL, considered as the mild deficiency of vitamin D. The CV of this level was calculated was 43.23% showed greater variation (Table 5).

### Depression and vitamin D profile of 61 years and above subjects (Group III)

In the 3rd group, 20 healthy and 16 subjects with depression of 60 years and above were selected (Table 6). For the 20 healthy subjects, mean ± SEM of age was found as 65.65 ± 1.06, while the mean ± SEM of vitamin D calculated was 64.09 ± 11.96 and 7.2% and 83.5% was found as a CV in age and vitamin D level, respectively (Table 6).

**Table 6:** Age vitamin D level of normal and depressed subjects of 61 years of age and above belonging to (Group III) given as the Mean ± SEM

S. No	Parameters	Normal subjects		Depressed subjects			*p* value	*p* value
		Age	Vitamin D (ng/mL)	Age	Vitamin D (ng/mL)	BDI score	Age	Vitamin D
1	Mean	65.65	64.09	66.25	21.8	30.94	> 0.05	< 0.05
2	Standard deviation	4.73	53.51	4.90	6.84	5.14		
3	Standard error of mean	1.06	11.96	1.22	1.7	1.3		
4	Coefficient of variance (%)	7.2	83.5	7.4	13.4	16.61		

*P* ≤ 0.05 = significant; *P* > 0.05 = non-significant

All the 16 subjects with depression were analyzed on the BDI scale in which 4 subjects were found with moderate depression, and twelve were having severely depression. Among the subjects with moderate depression, a vitamin D level of 1 was severe deficient, two were mildly deficient, and a vitamin D level of 1 was found normal (35.0 ng/mL). In the remaining 12 subjects with severe depression, the vitamin D level of 8 subjects was found to be mildly deficient, and three were severely deficient. While one subject showed a normal level of vitamin D. Here, an important correlation was observed between vitamin D levels and depression.

Statistical analysis of subjects shows the mean ± SEM of age 66.25 ± 1.2, while the mean ± SEM of vitamin D level was 21.8 ± 1.7in subjects having depression of age 60 and above. This value is very close to the severe deficiency of vitamin D level. On the other hand, on the BDI scale, 75% of the patients were with severe depression.

Furthermore, the mean ± SEM of BDI score was found to be 30.94 with a ± 5.14 standard deviation, which is the lower margin of severe depression. It is to be noted here that 13.4% variations from the mean vitamin D level suggest a more significant deficiency of vitamin D in subjects with depression in this case. Similarly, 16.6% variation from the mean BDI score was observed, which showed a strong correlation between vitamin D and depression.

Table 6, showing *p* value of age (*p* > 0.05) and *p* value of vitamin D (*p* < 0.05), *p* value of age is non-significant, while vitamin D is significant. Table 7 shows the vitamin D profile of all 100 normal subjects in which 59 (59%) subjects were male, and 41 (41%) female subjects irrespective of their age. Out of 100 normal subjects, 38 (38%) had normal vitamin D levels, of which 23 (60.5%) were female and 15 (39.5%) were male subjects. While 36 (36%) subjects had mild vitamin D levels, in which 6 (16.7%) were female, and 30 (83.3%) were male subjects. In Table 6 (17%), subjects had severe vitamin D deficiency, in which 6 (35.3%) were female, and 11 (64.7%) were male subjects. In all total 100 subjects, 9 (9%) showed a toxic level of vitamin D, of which 6 (66.7%) were female, and 3 (33.3%) were male.

**Table 7:** Vitamin D level of normal, depressed and depression status of depressed subjects has been mentioned as the mean ± SEM

		Vitamin D level							Depression level		
S.No		Normal subjects			Depressed subjects				Depressed subjects		
		Total	Male	Female	Total	Male	Female	Status	Total	Male	Female
1	Status	100	59	41	100	41	59		100	41	59
2	Normal level	38	15 (40)	23 (60)	19	07 (37.0)	12 (63.0)	Mildly depressed	26	16 (62.0)	10 (38.0)
3	Mildly deficient	36	30 (83.0)	06 (17)	51	27 (53.0)	24 (47.0)	Moderately depressed	24	08 (33.0)	16 (67.0)
4	Severely deficient	17	11 (65)	06 (35)	30	07 (23.0)	23 (77.0)	Severely depressed	50	17 (34.0)	33 (66.0)
5	Toxic level	09	03 (33.0)	06 (67)	–	–	–		–	–	–

Table 7 shows the vitamin D profile of all 100 subjects with depression, of which 59 (59%) subjects were female and 41 (41%) male subjects irrespective of their age. In all 100 subjects, 19 (19%) showed normal vitamin D levels, of which 12 (63.2%) were female, and 7 (36.8%) were male subjects. While 51 (51%) showed a mild deficiency of vitamin D level, in which 24 (47.1%) were female, and 27 (52.9%) were male subjects. In the same table 30 (30%) subjects were having severe vitamin D deficiency, in which 23 (76.7%) were female, and 7 (23.3%) were male subjects.

The depression profile of all 100 subjects with depression irrespective of their age. Table 7 indicates that out of 100 subjects, 26 (26%) were having mild depression, of these 26 subjects were having mild depression 10 (38.5%) were female, and 16 (61.5%) were male subjects. While 24 (24%) subjects were having moderate depression, in which 16 (66.7%) subjects were female, and 8 (33.3%) were male. In the same table, 50 (50%) subjects were having severe depression, in which 33 (66%) subjects were female, and 17 (34%) were male.

## Discussion

Vitamin D is a steroid hormone that performs various essential roles in the body, including its psychological roles [47]. Pakistan is in a Tropical region; hence sunlight is received by the people insufficiently amount, and people mostly avoid indoor activities. Many people living in Pakistan is unaware of the importance of the natural existence of vitamin D in sunlight and in daily food intake. Such factors should be taken into consideration. Vitamin D is known for its main function, which is the regulation of calcium and phosphorus concentration in the bones to facilitate cellular, neuromuscular, and ossification function [47]. Vitamin D deficiency can cause rickets in children, while osteomalacia in adults, osteoporosis, cancer, diabetes and autism results from vitamin D deficiency [37]. Treating vitamin D deficiency and the diseases such as cancer, multiple sclerosis, cardiovascular diseases require vitamin D supplements [9]. The Canadian guidelines for recommended dietary intake of vitamin D suggest 600 International Units for individuals aged 1 to 70 years and 800 IU for 71 years of age and above [53].

Hoogendijk et al. proposed the possible role of vitamin D in the brain [29]. Vitamin D is one of the secosteroid hormones that can pass through the blood–brain barrier (BBB), and its receptors are located in different regions of the brain, such as the cortex cerebellum and limbic system. It is proposed that a low level of vitamin D can lead to the elevation of PTH, which is associated with depression. Mainly, depression can be treated by treating hyperthyroidism [28].

Vitamin D deficiency has been observed in developed and developing countries including the US and Europe [38]. In the US, 36% of the adults with vitamin D deficiency has been observed [42]. In Australia, 1 in every 3 people has vitamin D deficiency [15]. In Pakistan, the prevalence of vitamin D deficiency in children is serious as 41% [16]. A study conducted in Faisalabad to determine vitamin D level reported that an epidemic in females as 87% of the pregnant females were found with vitamin D deficiency [4]. In another study, 90.1% of premenopausal females were presented with vitamin D deficiency in Karachi [30].

In this study vitamin D levels were noted within normal range in healthy subjects, while below normal ranges were observed in depressed subjects (Table 3). The study has reported some of the higher frequency of vitamin D deficiency in Peshawar region, with symptoms of depression. Significantly [32]. Patients with severe deficiency were above 70 years of age and more likely had mood disorder. Vitamin D deficiency has been concerned with anxiety and depression, although different researches have shown varied results. A strong association studies by Bernard and Hoogendijk demonstrated in elderly, hypovitaminosis D as a risk factor for depressive disorder in population-based cohorts [27]. Our results are in consistency that adults aged 20 or older did not find any relationship between hypovitaminosis D and depression as in group I.

The high prevalence of vitamin D deficiency in females might be, because they are not exposed to sunlight properly and are mostly housewives involved maximum time in domestic work. Females mostly wear hijab and burqa, due to which they are less exposed to the sunlight. The lack of awareness about a healthy lifestyle and lack of cooking expertise are contributing factors to the prevailing vitamin D deficiency. The pledge of government towards society is required to combat the condition. A national program on vitamin D supplementation and a public awareness campaign is urgently needed [30]. The results of this study indicated that vitamin D deficiency is associated with an increased risk of depression in isolated subjects.

Depression and other mood disorders are major health concerns throughout the world. It sheds very negative impacts on the quality of life, and it has been estimated that by 2020 depression will be ranked as second in global health burdens to the heart diseases [33]. Depression is characterized as persistent low mood, sadness, and hopelessness with pessimism. The effectiveness of depression sometimes ends their life, committing suicide [33].

Vitamin D deficiency is linked with the development of mood disorders by Stone and colleagues in 2012. It was proposed that vitamin D supplements may play a role in the treatment of depression [48]. However, low levels of vitamin D may be contributed to uncontrolled mood and behavior. The deficiency can damage brain health and thus cognitive function.

People with depression live a very disturbing life. They feel tired and thus are unable to perform their routine life activities. Their family life is also disturbed, and their vitamin D levels is low [50]. In the US, depression is considered a leading cause of disability in the young population [48].

This study allowed to understand the combination of anti-depressant and psychotherapy used as a conventional treatment for depression during vitamin D deficiency [50]. The exercise can be an effective intervention in the reduction of depressive symptoms. Complementary and alternative medical interventions for depression include omega-3 essential fatty acids, tryptophan, adenosylmethionine, folic acid, vitamin B12, and zinc [13]. Therefore, the prevalence of vitamin D deficiency and its multi-system implications, the serum calcium level, and phosphorus do not predict its deficiency because of the limited studies available in Pakistan. Further implications must be applied to initiate the advanced research work.

## Conclusions

The study showed a very high frequency of vitamin D deficiency in the subjects with depression in Peshawar, Pakistan. Vitamin D deficiency was observed more in females as compared to males. In this study, the correlation between vitamin D and depression was tried to ascertain, and it has been observed that prevalence of depression is common in those individuals who have a low level of vitamin D. The growing data of vitamin D deficiency in Pakistan may appear surprising, since we receive ample sunshine almost all year round.

### Limitations of our study

A limitation is the progress of the cohort of this study. Since, subjects included were vitamin D deficient. As compared to the general population, there should be potential bias for indicative patients with low vitamin D level. In addition, due lack of data, we could not able to determine differences of ages with vitamin D levels. However, our study might help further to analyse the degree of vitamin D deficiency and its association with anxiety and major depressive disorder.

## Supplementary Information

**Additional file 1: Table S1.** Reference Ranges for 25 (OH) D (Ferrari, Lombardi, & Banfi, 2017).

## Data Availability

All the data is contained in the manuscript.

## Abbreviations

BDI: Beck depression inventory
CV: Coefficient of variation
SEM: Standard error
