# Supplementary material for: Vitamin D status and its correlation to depression
Source: Ann Gen Psychiatry. 2022 Aug 18;21:32. doi: 10.1186/s12991-022-00406-1 (PMC9389668; doi:10.1186/s12991-022-00406-1)
Supplement: Supplementary file 1 — Additional file 1: Table S1. Reference Ranges for 25 (OH) D (Ferrari, Lombardi, & Banfi, 2017). [file 12991_2022_406_MOESM1_ESM.docx]

**Supplementary File S1**

**Vitamin D Status and its Correlation to Depression**

Bashir khan^1^, Huma Shafiq^2^, Seyyedha Abbas^1^, Summeira Jabeen^1^, Sikandar Ali Khan^1^, Tayyaba Afsar^3^, Ali Almajwal^3^, Nawaf W. Alruwaili^3^, Dara al-disi^3^, Sultan Alenezi^2^, Zahida Parveen^4^, Suhail Razak^3^

**Table 1:** Reference Ranges for 25 (OH) D (Ferrari, Lombardi, & Banfi, 2017)

| **S.No** | **Nmol/L** | **ng/ml** | Health Status |
| --- | --- | --- | --- |
|  |  |  |  |
| 1 | <30 | <12 | Causing Rickets in Children, Oteomalacia in adults |
|  |  |  |  |
| 2 | 30-50 | 12-20 | Deficient for normal bones functions |
|  |  |  |  |
| 3 | ≥50 | ≥20 | Sufficient level for bones |
|  |  |  |  |
| 4 | >125 | >50 | The toxic level of vitamin D causes harmful effects. |
|  |  |  |  |

**Table 2.:** Scoring of Depression according to Allen (Allen et al., 2007)

| **S.NO** | **Score** | **Level of Depression** |
| --- | --- | --- |
|  |  |  |
| 1 | 0-10 | Normal |
|  |  |  |
| 2 | 11-16 | Mild mood disturbance |
|  |  |  |
| 3 | 17-20 | Border line clinical depression |
|  |  |  |
| 4 | 21-30 | Moderate depression |
|  |  |  |
| 5 | 31-40 | Severe depression |
|  |  |  |
| 6 | 41-63 | Extreme depression |
|  |  |  |

**References**

Allen, C. K., Austin, S. L., David, S. K., MHE, O., McCraith, D. B., & Riska-Williams, L. J. J. O. T. (2007). Manual for the Allen cognitive level screen-5 (ACLS-5) and Large Allen cognitive level screen-5 (LACLS-5). *56*, 609-639.

Ferrari, D., Lombardi, G., & Banfi, G. J. B. m. (2017). Concerning the vitamin D reference range: Pre-analytical and analytical variability of vitamin D measurement. *27*(3), 453-466.
